# Plate-augmented fixation of comminuted Letenneur type II Hoffa fractures provides favorable stability compared to isolated posteroanterior screw fixation – a biomechanical study

**DOI:** 10.1007/s00402-025-05920-x

**Published:** 2025-05-19

**Authors:** Christian Peez, Moritz F. Lodde, Ivan Zderic, R. Geoff Richards, Ludmil Drenchev, Hristo K. Skulev, Boyko Gueorguiev, Christoph Kittl, Michael J. Raschke, Elmar Herbst

**Affiliations:** 1 Department of Trauma, Hand and Reconstructive Surgery, University Hospital Münster, Münster, Germany; 2 AO Research Institute, Davos, Switzerland; 3 Bulgarian Academy of Sciences, Institute of Metal Science, Equipment and Technology with Chydroaerodynamic Centre “Acad. A. Balevski”, Sofia, Bulgaria

**Keywords:** Hoffa fracture, Letenneur classification, Posteroanterior screw fixation, Plate-augmented fixation, Biomechanical testing, Motion tracking

## Abstract

**Introduction:**

Current literature lacks recommendations regarding proper fixation of comminuted coronal plane fractures of the posterior femoral condyles (Hoffa fractures). Therefore, the aim of this study was to compare the biomechanical characteristics of different plate-augmented constructs to isolated posteroanterior screw fixation in comminuted lateral Hoffa fractures.

**Materials and methods:**

Comminuted Letenneur type IIb lateral Hoffa fractures were simulated in 24 human cadaveric distal femora. The fractures were fixed with either isolated crossed posteroanterior screws or additionally with either a posterior plate, a lateral locking plate, or combined posterior and lateral locking plates. All specimens were biomechanically tested under progressively increasing cyclic loading until failure, while capturing the interfragmentary movements of the Hoffa and intercalary fragments via motion tracking.

**Results:**

Plate-augmented posteroanterior screw fixation of the Hoffa fragment exhibited higher cycles to failure, higher failure loads (*p* < 0.05) and less axial displacements (*p* < 0.05) compared to isolated posteroanterior screw fixation. Both additional lateral locking and double plate fixations of the intercalary fragment provided higher cycles to failure, higher failure loads (*p* < 0.05) and less axial displacement (*p* < 0.05) than isolated posteroanterior screw fixation, whereas additional posterior plate fixation did not significantly increase cycles to failure, failure loads and axial displacement (n.s.). Fracture gap twisting and opening did not differ significantly between the four constructs for the Hoffa fragment, while plate-augmented constructs provided less fracture opening of the intercalary fragment compared to isolated posteroanterior screw fixation (*p* < 0.01).

**Conclusions:**

Plate-augmented posteroanterior screw fixation of comminuted Letenneur type IIb Hoffa fractures provided greater biomechanical stability than isolated posteroanterior screw fixation. While additional lateral or double plate fixation improves the stability of both the intercalary and Hoffa fragment, posterior plating stabilized only the Hoffa fragment.

## Introduction

Being characterized by a coronal shear fracture of one or both posterior femoral condyles [4, 16], Hoffa fractures represent a rare but devastating injury to the knee joint, accounting for up to 13% of distal femur fractures [10]. Treatment of these fractures remains challenging as demonstrated in clinical studies reporting unsatisfactory functional outcomes [6, 13, 15, 18, 32, 35]. Key factors for improving patient-reported outcomes and reducing the risk of posttraumatic osteoarthritis are anatomic reconstruction of the articular surface and preservation of limb alignment, which requires a proper fracture reduction and fixation [7, 19].

Screw fixation represents the most common internal fixation method of Hoffa fractures [2, 6, 22, 42], while various plate-augmented constructs have been recently suggested to improve neutralization of vertical shear forces. However, the position of the plates may vary depending on the proposed biomechanical principles to ensure absolute stability and avoid impaired fracture healing [25–27, 33]. In this context, the biomechanical stability of lateral or posterior plate fixation of large Hoffa fragments (Letenneur type I) has been investigated with favorable results versus isolated anteroposterior and posteroanterior screw fixation [27, 33]. However, in lateral Hoffa fractures, smaller Letenneur type II fragments are more frequently observed, being commonly associated with comminution zones in the weight-bearing area of the femoral condyles [38]. Up to date, there is a lack of evidence regarding optimal fixation techniques to properly address these special Hoffa fracture subtypes with intercalary fragments.

Therefore, the aim of this study was to compare the biomechanical characteristics of different plate-augmented constructs to isolated posteroanterior screw fixation in comminuted Letenneur type II lateral Hoffa fractures. It was hypothesized that augmented plate constructs would demonstrate favorable stability of both the Hoffa fragment and the intercalary fragments compared to an isolated posteroanterior screw fixation.

## Materials and methods

Twenty-four fresh-frozen non-paired human cadaveric knees from 8 female and 16 male donors aged 73.7 (10.9) years (mean (standard deviation, SD)) (range 46–85 years) were obtained from an international tissue bank (Science Care, Phoenix, Arizona, USA). In a written consent, all donors bequeathed their corpse for use in medical science during their lifetime.

The distal femora of all knees were assessed for bone mineral density (BMD) within the trabecular region of the lateral femoral condyle using computed tomography (CT) scanning (Revolution EVO, General Electric Healthcare, Buckinghamshire, UK). A phantom (BDC-6, QRM GmbH, Möhrendorf, Germany) was subsequently analyzed using an image processing software (Amira, v.6.0, Thermo Fisher Scientific, Waltham, MA, USA) with segmentation between 150 and 450 mgHA/cm^3^. Based on BMD, the knees were assigned to four groups consisting of six specimens each (*n* = 6), with a homogenous BMD distribution between the four groups (mean difference [∆] – 2.4 mgHA/cm^3^, 95% confidence interval [CI] – 60.7-55.8 to ∆ 7.8 mgHA/cm^3^, -50.5–66.1).

### Specimen Preparation

**Fig. 1:**
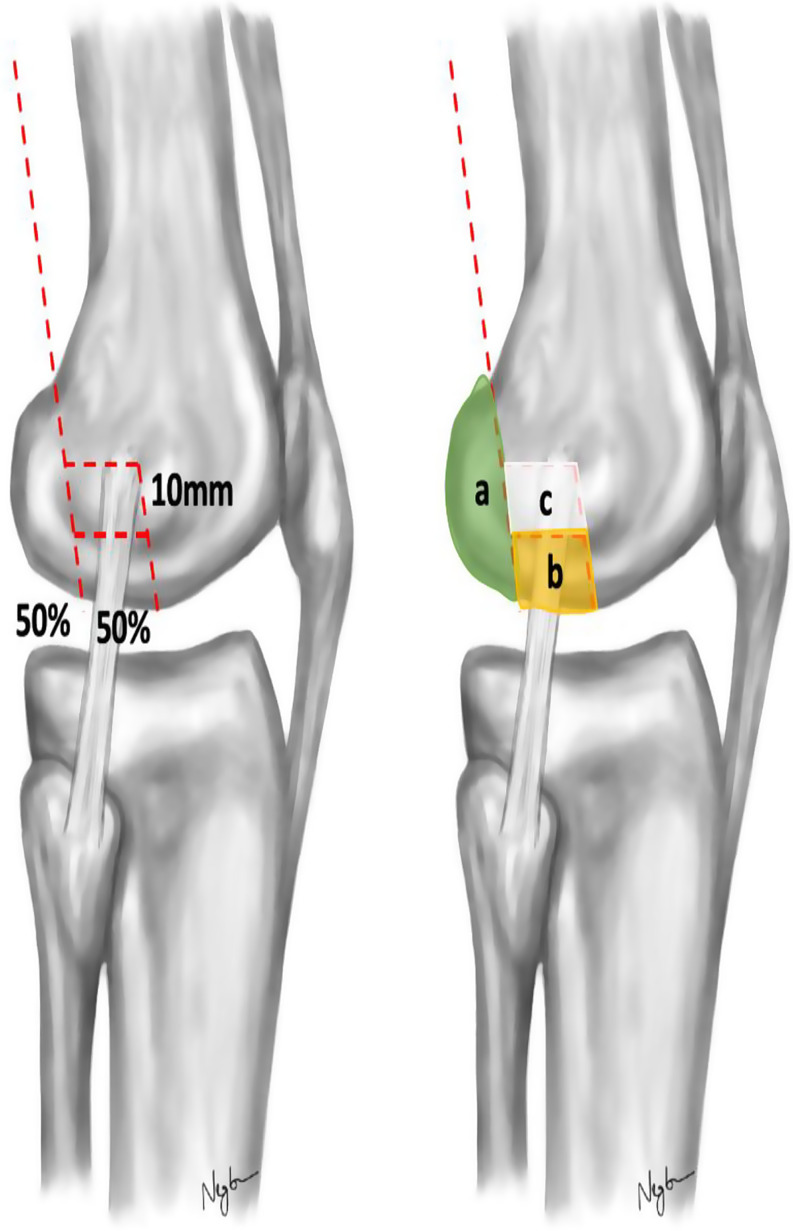
Schematic illustration of the fracture model. A Letenneur type IIb Hoffa fragment (**a**,** green**) and an intercalary fragment in the weight-bearing area of the distal femur (**b**,** yellow**) with a defect zone (**c**,** white**) were created to simulate comminuted lateral Hoffa fractures

**Fig. 2:**
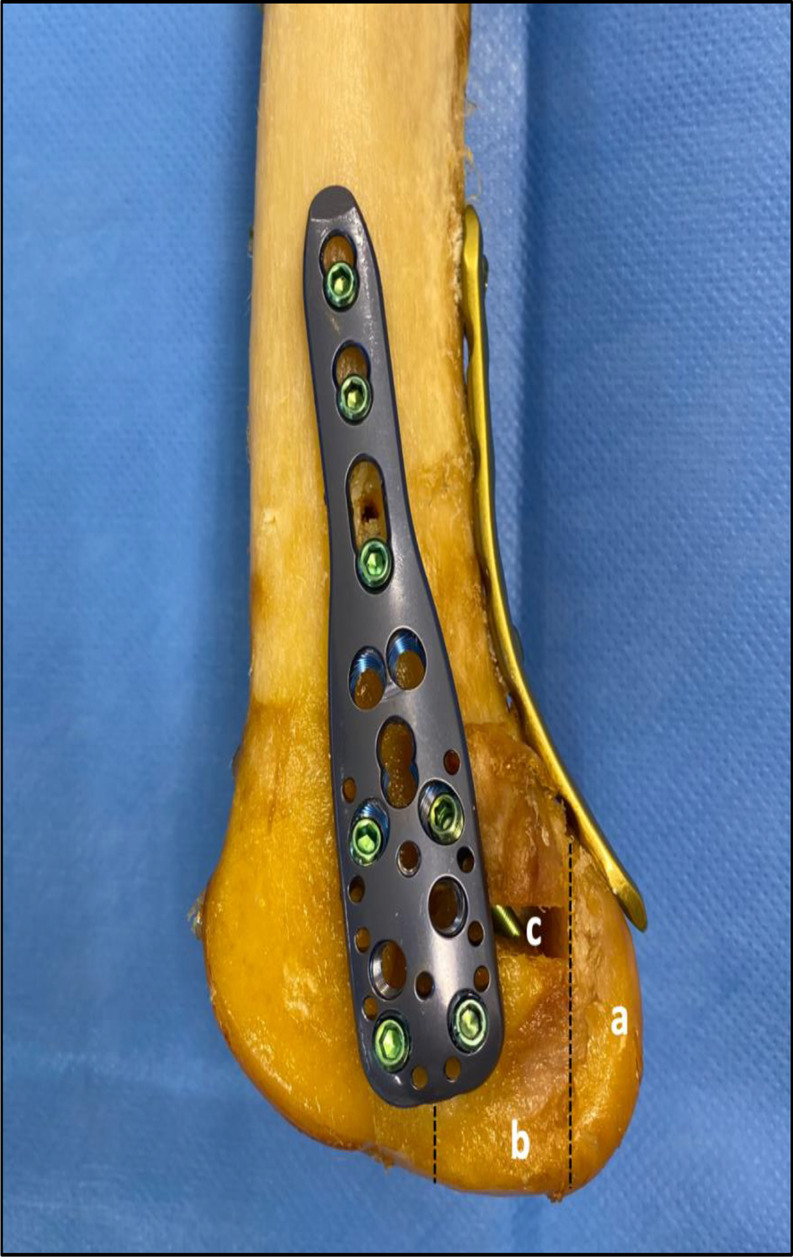
Exemplified photograph of a left distal femur after augmented double plate fixation of the comminuted Letenneur type IIb Hoffa fractures. A Letenneur type IIb Hoffa fragment (**a**) and an intercalary fragment in the weight-bearing area of the distal femur (**b**) with a defect zone (**c**) were created to simulate comminuted lateral Hoffa fractures

Prior to preparation and biomechanical testing, the knees were thawed at room temperature for 24 h. After cutting the distal femur 250 mm proximal to the joint line, the knee joints were disarticulated to harvest the distal femur. Once the entire soft tissue was removed, lateral Hoffa fractures were simulated according to the Letenneur classification [11]. First, the anteroposterior diameter of the lateral femoral condyle was determined from its posterior border to the tangent of the posterior femoral cortex. Using an oscillating saw, an osteotomy was created in the coronal plane of the posterior lateral femoral condyle parallel to the posterior cortex, creating a Letenneur type IIb fracture with a Hoffa fragment involving 50% of the anteroposterior length of the lateral femoral condyle [11]. Then, an additional coronal osteotomy parallel to the posterior cortex and an axial osteotomy parallel to the axis of the distal femoral epicondyles were performed to create an intercalary fragment in the weight-bearing area of the lateral distal femur involving the remaining 50% of the anteroposterior diameter of the lateral posterior femoral condyle. A 10 mm bone block proximal to the intercalary fragment was then removed to simulate a comminuted Letenneur type IIb lateral Hoffa fracture [38] (Figs. 1 and 2).

**Fig. 3:**
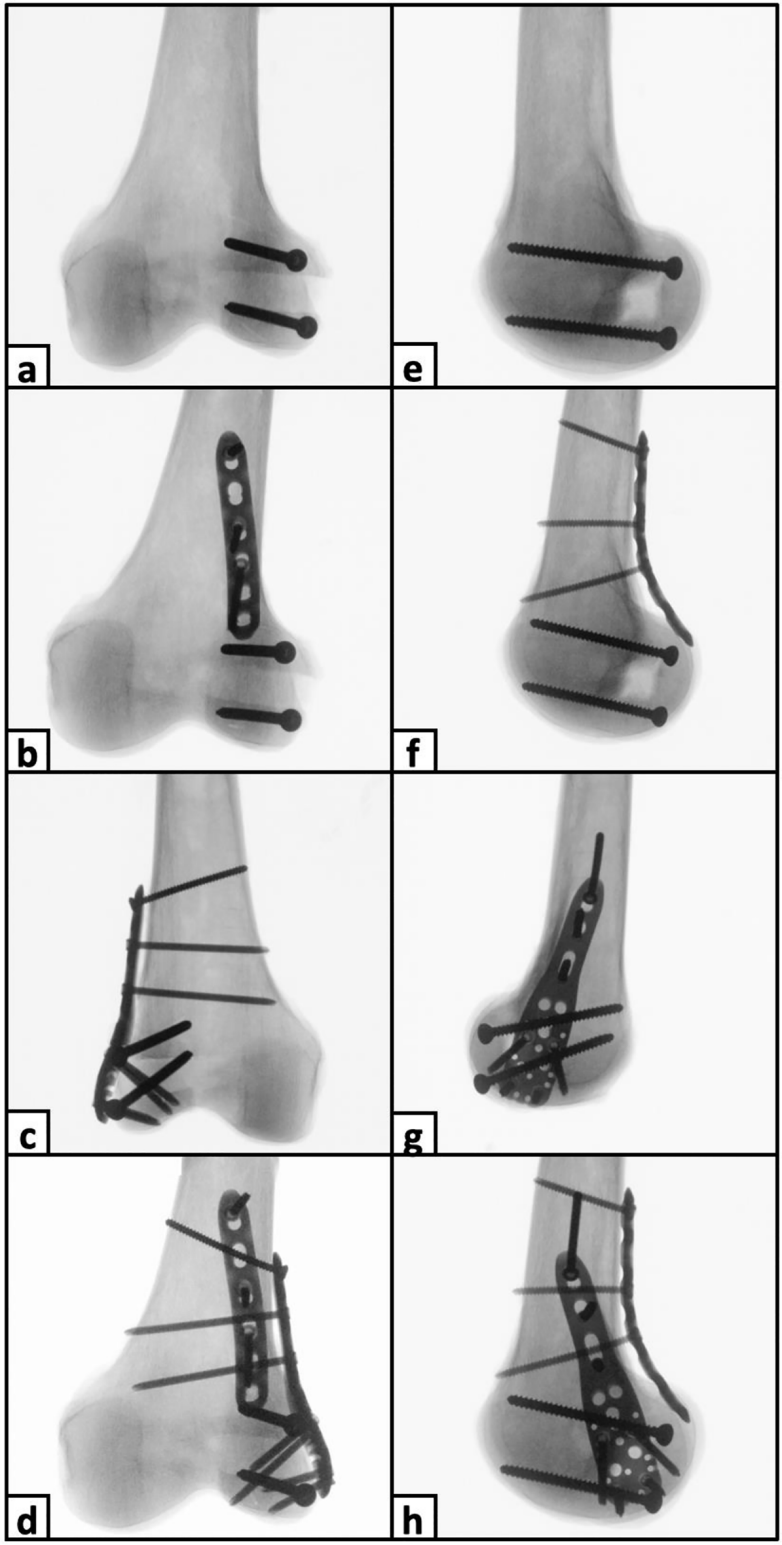
Anteroposterior (**a - d**) and mediolateral (**e - h**) radiographs of specimens following crossed posteroanterior screw fixation (**a**,** e**) as well as additional posterior plate fixation (**b**,** f**), lateral locking plate fixation (**c**,** g**), and double plate fixation (**d**,** h**)

Depending on the group assignment, the fragments were fixed after anatomic fracture reduction with either isolated crossed posteroanterior screws (PA screws) or additionally with either a posterior plate (PA screws + posterior plate), lateral locking plate (PA screws + lateral plate), or combined posterior and lateral locking plates (PA screws + double plate). For crossed PA screw fixation, two parallel 4.5 mm fully-threaded cortical screws (Johnson & Johnson MedTech, Zuchwil, Switzerland) were inserted beneath the posterolateral border of the articular cartilage of the condylar fragment, both aiming 45° anteriorly and 10° proximally [24] (Fig. 3a and e). Additional posterior plate fixation was performed using a 3.5 mm 6-hole locking compression plate (LCP) (Johnson & Johnson MedTech, Zuchwil, Switzerland). The plate was secured bicortically with three 3.5 mm self-tapping cortical screws and was slightly pre-contoured to buttress the posterior femoral condyle while fastening the screws (Fig. 3b and f). Additional lateral locking plate fixation was performed using a 3.5 mm 3-hole PHILOS plate (Johnson & Johnson MedTech, Zuchwil, Switzerland), as in the clinical experience the plate may fit the anatomy of the distal lateral femur. In row A and C holes four 3.5 mm self-tapping locking screws reaching but not penetrating the second cortex were inserted, while the shaft holes were secured with three 3.5 mm self-tapping bicortical cortical screws (Fig. 3c and g). For additional double plate fixation, posterior plate and lateral locking plate fixation were used in combination as described above (Fig. 3d and h). Upon completion of the surgical procedures, the proximal 6 cm of the femora were embedded in a polymethylmethacrylate (PMMA, Suter Kunststoffe AG, Fraubrunnen, Switzerland) socket. Finally, retro-reflective marker sets were attached to the femoral shaft, the Hoffa fragment, and the intercalary fragment for motion tracking.

### Biomechanical testing

**Fig. 4:**
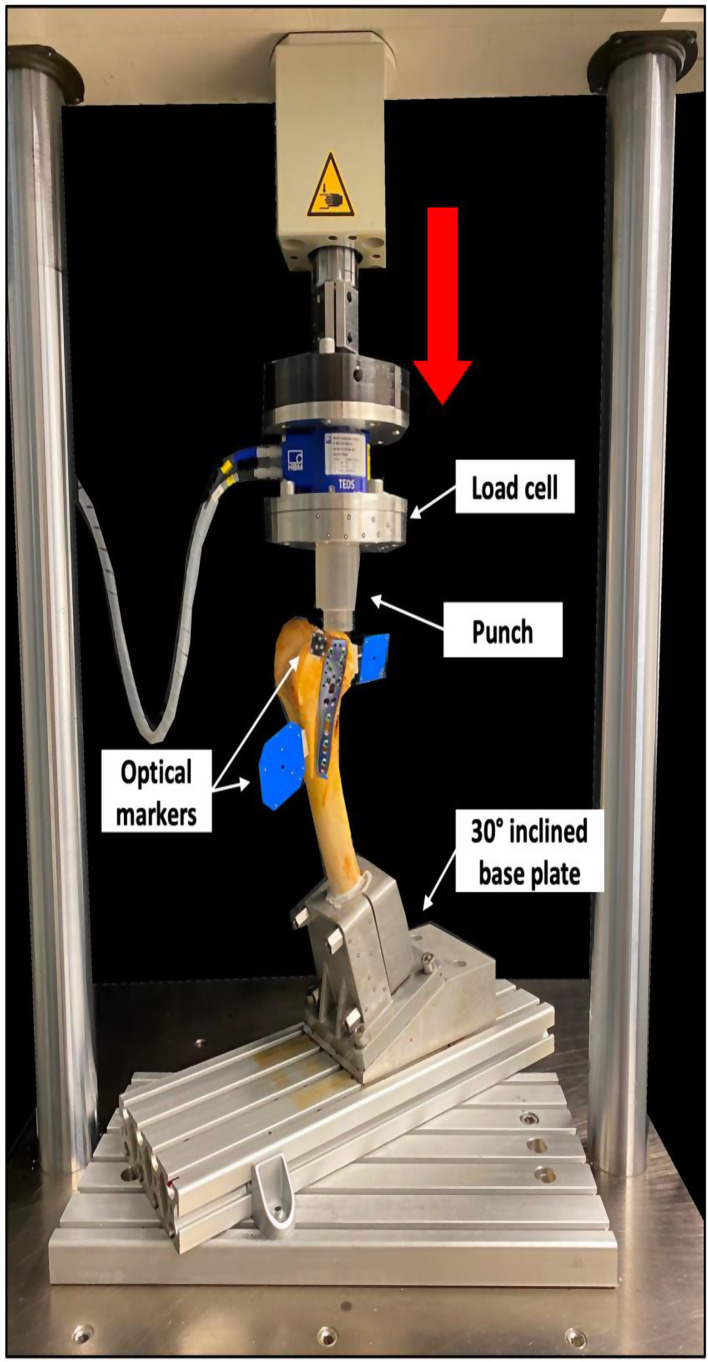
Setup with a specimen mounted for biomechanical testing

Biomechanical testing was performed using a servo-hydraulic materials testing machine (Bionix 858.20, MTS Systems Corp., Eden Prairie, MN, USA) equipped with a 5 kN load cell (HBM, Darmstadt, Germany) that allows position and force control with 0.05% accuracy. As previously described [24], each specimen was tested in an inverted upright standing position. For this purpose, each femur was rigidly mounted to the materials testing machine base via an aluminum base plate inclined at 30° in the sagittal plane to simulate axial loading of the Hoffa fragments at 30° knee flexion. Axial compression along the machine axis was applied via a custom-made PMMA punch allowing a homogenous load transfer to the hemispherical surface of the lateral posterior femoral condyle (Fig. 4).

Starting from a preload of 20 N, axial loading commenced with a non-destructive quasi-static ramp to 100 N at a rate of 20 N/sec, followed by a progressively increasing cyclic loading at 2 Hz. While maintaining a constant valley load of each cycle at 20 N, the peak load increased monotonically from 100 N at a rate of 0.1 N/cycle until reaching the limit of 10 mm actuator displacement.

### Data acquisition and evaluation

Actuator displacement and axial compression forces were recorded by the test system controllers of the testing machine at 128 Hz. Based on these data, force-displacement curves were generated to calculate the construct stiffness, defined as the slope of the initial quasi-static ramp within the linear loading range between 20 and 100 N.

In addition, a stereographic optical motion tracking system using contactless full-field deformation technology (Aramis SRX, Carl Zeiss GOM Metrology GmbH, Braunschweig, Germany) continuously captured the interfragmentary displacement in all six degrees of freedom, operating at a maximum acceptance error of 0.004 mm [30]. Interfragmentary movements were evaluated at the initial stage and thereafter at 1000, 2000, 3000, 4000 and 5000 cycles under peak loading conditions with respect to the beginning of the cyclic test. Specifically, fracture site displacement along the femoral shaft axis, defined as axial displacement, was captured between the most articular margin of the Hoffa and intercalary fragment and the femoral shaft. Further, interfragmentary rotation around the mediolateral axis, defined as fracture gap opening, and around the anteroposterior axis, defined as fracture gap twisting, of both the Hoffa and intercalary fragments were evaluated with respect to the femoral shaft. As fracture step-offs greater than 2 mm were associated with increased articular pressures [17, 28] and therefore with early onset of osteoarthritis [8, 29, 34] following tibial plateau fractures, reaching an axial displacement of 2 mm was set as the clinical failure criterion for both fragments in intraarticular distal femur fractures. The number of cycles until its fulfillment under peak loading were defined as cycles to failure, followed by a calculation of the corresponding failure load for both the Hoffa and intercalary fragments.

### Statistical analysis

Statistical analysis was performed using Prism (Version 9, GraphPad Software, Boston, USA). Descriptive data is presented as mean value with standard deviation (SD), and differences between the groups – as mean differences with 95% confidence intervals. Normality of data distribution within each fixation technique was tested and proved using the Shapiro-Wilk test. Significant differences among the groups regarding BMD, construct stiffness, cycles to failure and failure load were detected with One-Way Analysis of Variance [14] and Tukey´s post hoc test for multiple comparison. Two-Way Repeated-Measures ANOVAs with Geisser-Greenhouse correction and post hoc Tukey´s multiple comparison test were conducted to identify significant differences among the groups with regard to axial displacement, fracture gap twisting and fracture gap opening evaluated over 5000 test cycles. Overall level of significance was set at *p* = 0.05.

An a-priori power analysis was performed using G*Power-2 software (University Düsseldorf, Düsseldorf, Germany) [5]. Based on means and standard deviations from a previous study evaluating the biomechanical performance of different screw configurations for fixation of Hoffa fractures [41], it was assumed that a sample size of 6 would allow the identification of changes in displacement of 0.2 mm with 95% power at the significance level of *p* = 0.05.

## Results

The mean BMD (mgHA/cm^3^) ranged from 151.7 ± 37.3 (mean value ± SD) to 159.5 ± 28.9, demonstrating a homogenous distribution among the testing conditions (*p* ≥ 0.98) (Table 1). Construct stiffness of additional posterior and lateral locking plate fixation was comparable to isolated crossed PA screw fixation (*p* ≥ 0.15), whereas an additional double plate fixation provided significantly higher stiffness (*p* < 0.05) (Table 1).

**Table 1:** Outcome measures for the investigated parameters of interest bone mineral density, construct stiffness, cycles to failure and corresponding failure loads, shown for each fixation technique/group separately in terms of mean value and standard deviation together with the corresponding *p*-value from the statistical comparison of the plate constructs versus isolated posteroanterior (PA) screw fixation

	PA screws	PA screws + Posterior plate (*p*-value)	PA screws + Lateral plate (*p*-value)	PA screws + Double plate (*p*-value)
**Bone Mineral Density [mgHA/cm** ^**3**^ **]**	151.7 ± 37.3	154.0 ± 32.4 (0.99)	159.5 ± 28.9 (0.98)	157.1 ± 43.9 (0.99)
**Construct stiffness [N/mm]**	425. 2 ± 147.7	623.8 ± 193.8 (0.15)	627.9 ± 144.7 (0.16)	699.9 ± 169.0 **(< 0.05)**
**Cycles to failure Hoffa fragment**	3514 ± 1927	9869 ± 4690 **(< 0.05)**	9981 ± 2562 **(< 0.05)**	13,868 ± 6019 **(< 0.01)**
**Failure load Hoffa fragment** **[N]**	551 ± 271	1087 ± 428 **(< 0.05)**	1098 ± 229 **(< 0.05)**	1326 ± 475 **(< 0.05)**
**Cycles to failure intercalary fragment**	3451 ± 1792	5889 ± 3291 (0.56)	9891 ± 2562 **(< 0.05)**	12,260 ± 4313 **(< 0.01)**
**Failure load intercalary fragment** **[N]**	541 ± 251	689 ± 300 (0.56)	1005 ± 295 **(< 0.05)**	1326 ± 475 **(< 0.05)**

### Letenneur type IIb Hoffa fragment

**Fig. 5:**
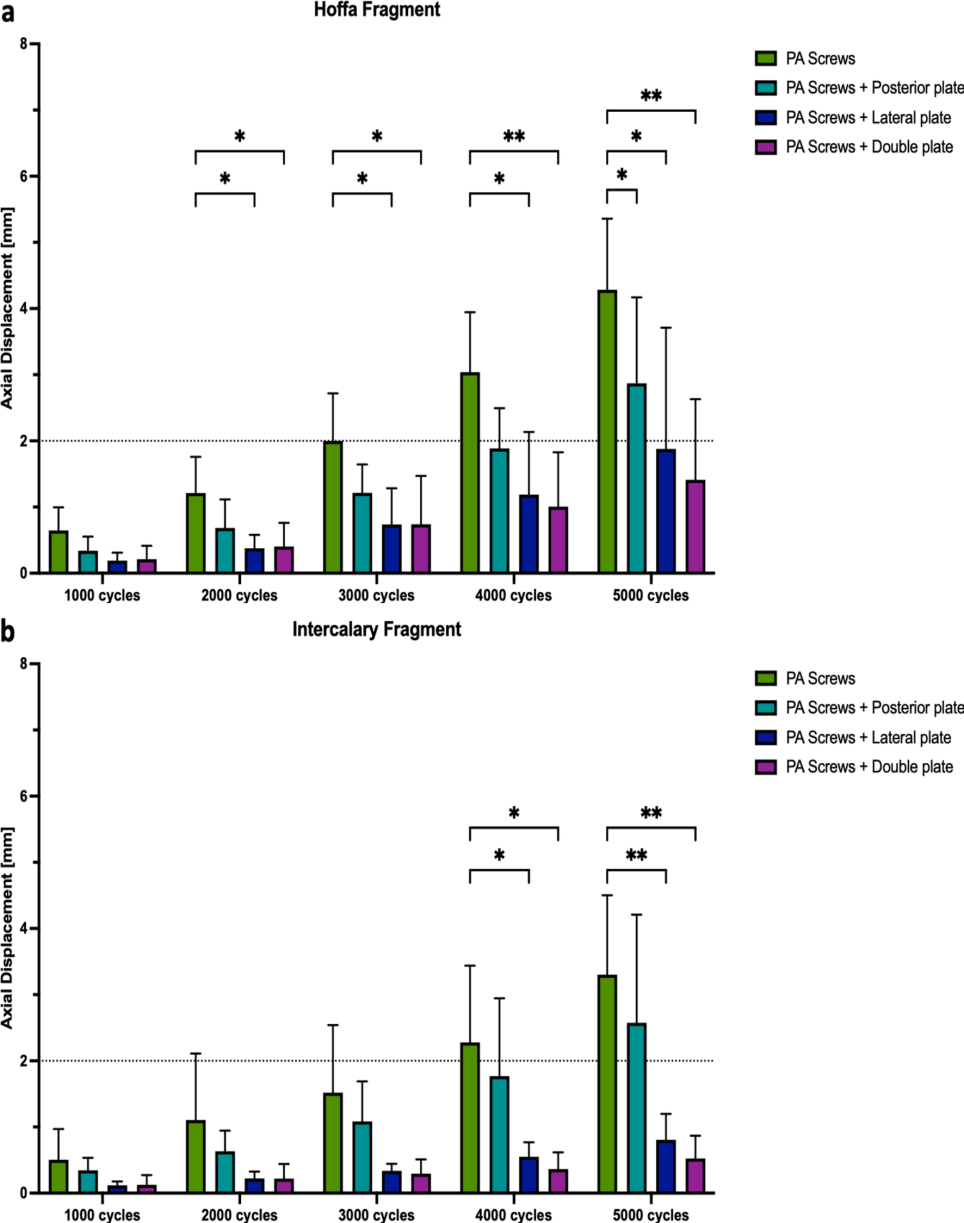
Axial displacement after 1000, 2000, 3000, 4000, and 5000 cycles, shown separately for each group as mean value and standard deviation, together with the corresponding p-value from the statistical comparisons between groups. **(a)** Hoffa fragment. **(b)** Intercalary fragment* = *p* < 0.05, ** = *p* < 0.01

**Fig. 6:**
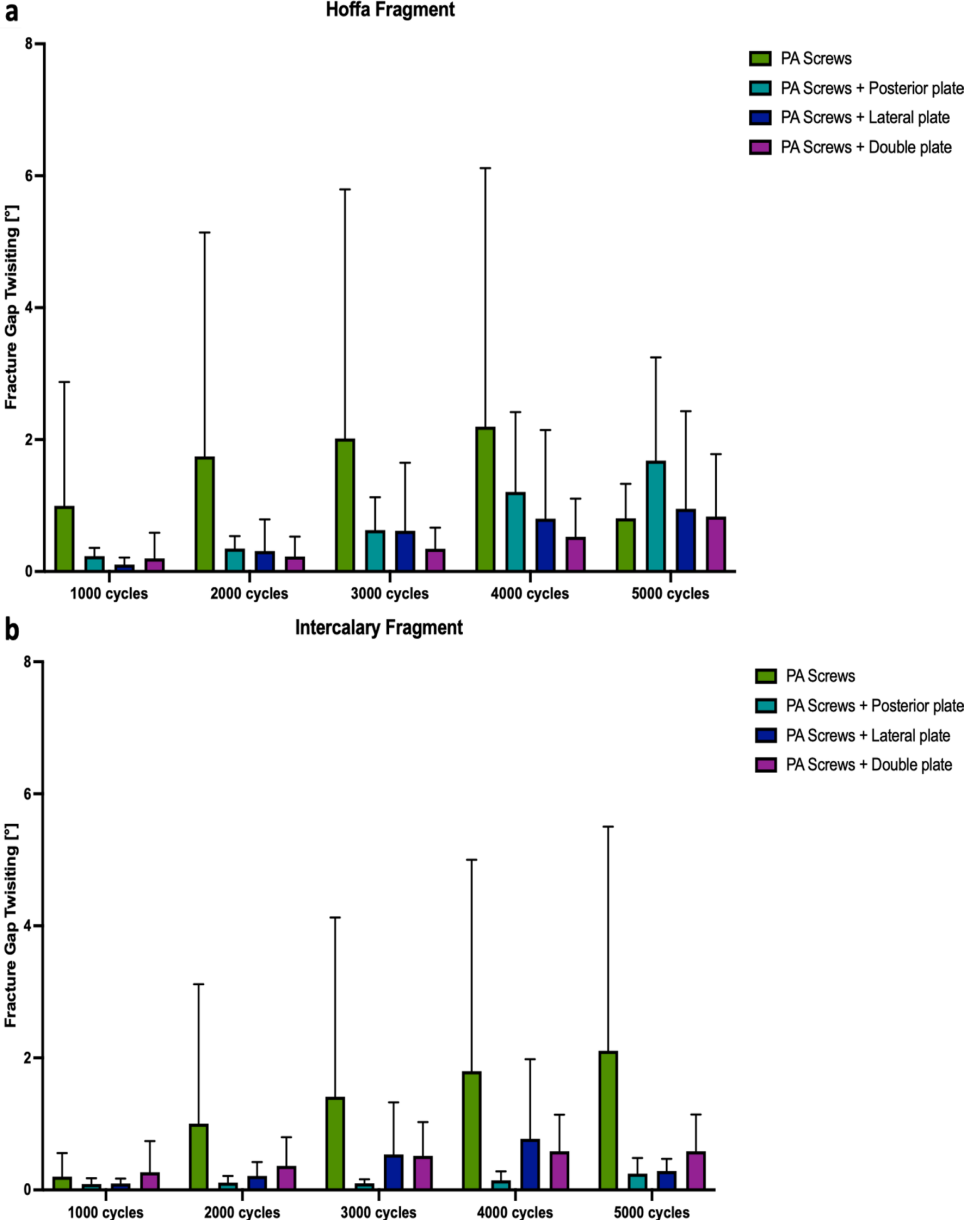
Fracture gap twisting after 1000, 2000, 3000, 4000, and 5000 cycles, shown separately for each group as mean value and standard deviation, together with the corresponding p-value from the statistical comparisons between groups. **(a)** Hoffa fragment. **(b)** Intercalary fragment

**Fig. 7:**
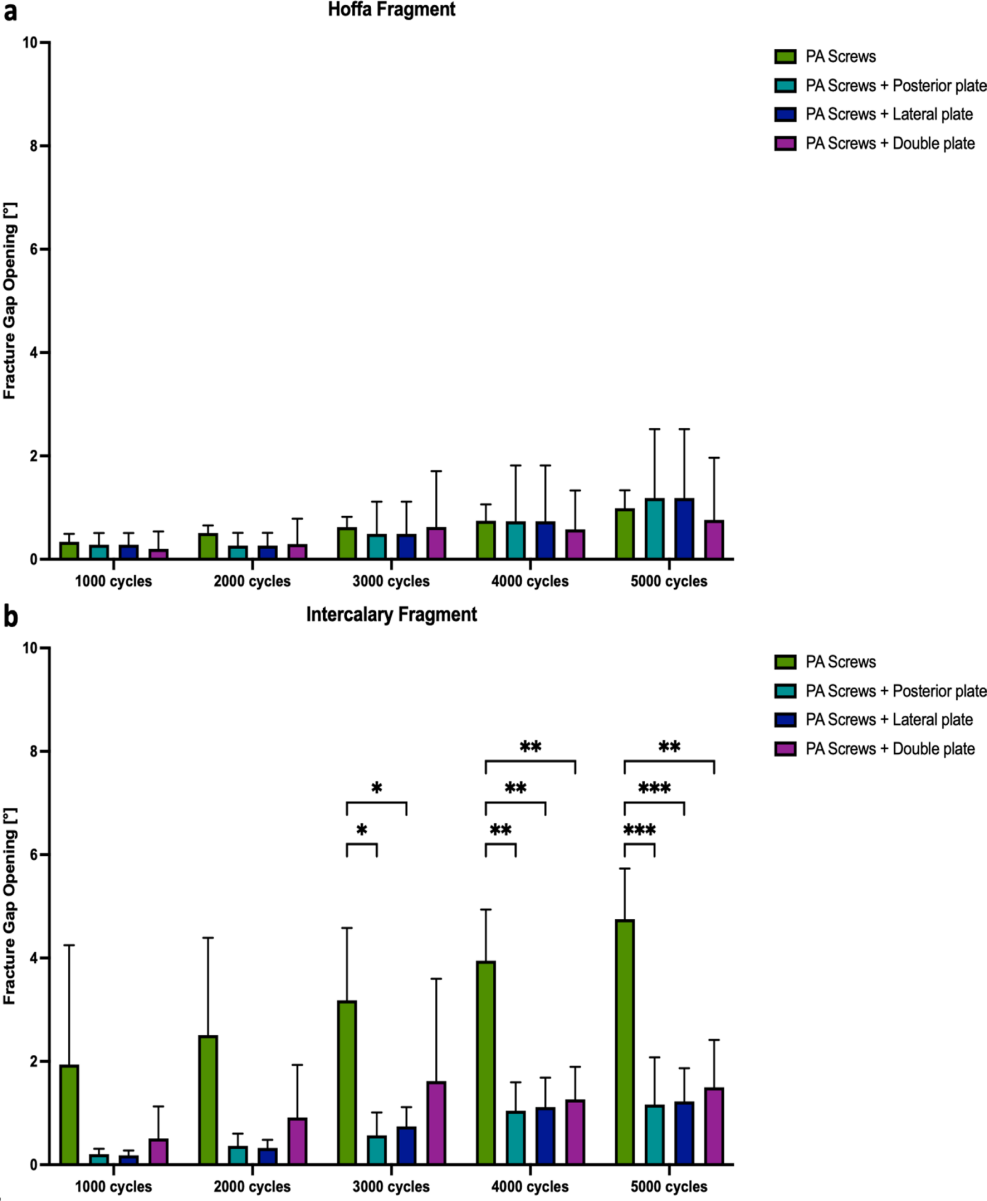
Fracture gap opening after 1000, 2000, 3000, 4000, and 5000 cycles, shown separately for each group as mean value and standard deviation, together with the corresponding p-value from the statistical comparisons between groups. **(a)** Hoffa fragment. **(b)** Intercalary fragment* = *p* < 0.05, ** = *p* < 0.01, *** = *p* < 0.001.

**Fig. 8:**
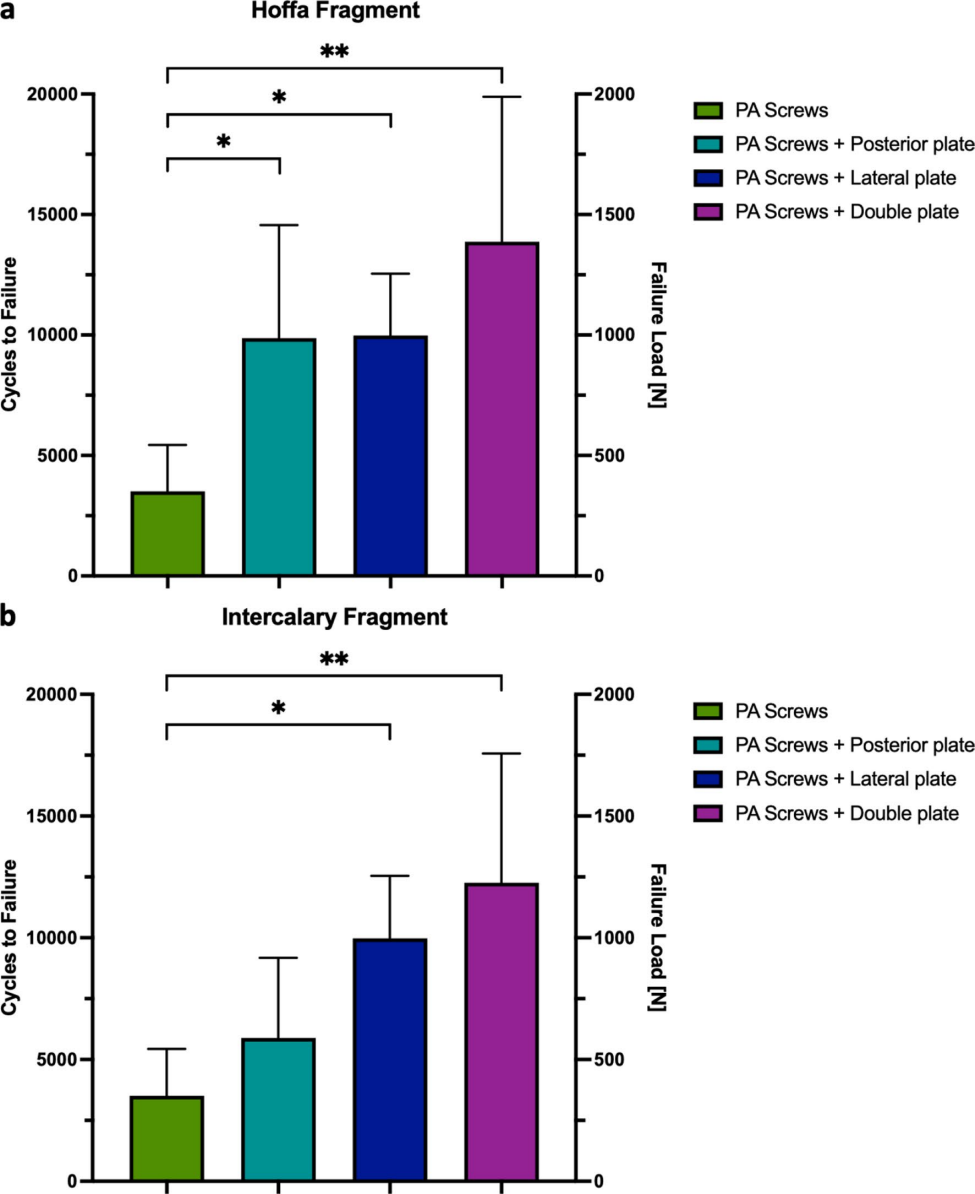
Cycles to failure and corresponding failure load following fixation of comminuted Letenneur type IIb lateral Hoffa fractures with either isolated crossed posteroanterior screws (PA screws) or additionally with posterior plate fixation (PA screws + Posterior plate), lateral locking plate fixation (PA screws + Lateral plate) or combined posterior and lateral locking plate fixation (PA screws + Double plate). Error bars indicate mean value ± standard deviation. * = *p* < 0.05, ** = *p* < 0.01.

Plate-augmented PA screw fixation of the Hoffa fragment exhibited significantly less axial displacement after 5000 cycles compared to isolated crossed PA screw fixation (*p* < 0.05) (Fig. 5a). During the 5000 cycles, fracture gap twisting and opening were not significantly different among the four study groups (*p* ≥ 0.22, Figs. 6a and 7a). Cycles to failure and corresponding failure loads were not significantly different between the plating groups (*p* ≥ 0.39), while isolated PA screw fixation resulted in earlier construct failure with significantly less cycles to failure and failure loads (*p* < 0.05) (Fig. 8a; Table 1).

### Intercalary fragment

Augmented lateral locking plate and double plate constructs exhibited significantly less axial displacement of the intercalary fragment after 4000 cycles compared to isolated crossed PA screw fixation (*p* < 0.05), whereas additional posterior plate fixation displayed comparable axial displacement over 5000 test cycles (*p* ≥ 0.59) (Fig. 5b). Over 5000 cycles, fracture gap twisting did not significantly differ between the groups (*p* ≥ 0.58), while all plate-augmented constructs provided significantly less fracture gap opening than isolated PA screw fixation (*p* < 0.01) (Figs. 6b and 7b). Additional lateral locking and double plate fixation of the intercalary fragment provided higher cycles to failure and failure loads compared to isolated PA screw fixation (*p* < 0.05). Augmented posterior plate fixation showed comparable cycles to failure and failure loads to isolated PA screw fixation (*p* = 0.56) (Fig. 8b; Table 1).

## Discussion

The most important finding of the present study was that plate-augmented posteroanterior screw fixation of comminuted Letenneur type IIb Hoffa fractures provided greater biomechanical stability than isolated posteroanterior screw fixation. Stability of the Hoffa fragment was improved with all three additional plating techniques, whereas the intercalary fragment was adequately fixed with either a lateral locking or a double plate fixation only.

Hoffa fractures are frequently associated with unsatisfactory functional outcomes [6, 13, 18, 32, 35], mainly attributed to the increased risk of posttraumatic osteoarthritis [18], non-union and avascular osteonecrosis [3, 12, 21]. In fact, Onay et al. [18] reported poor long-term results for surgically treated Hoffa fractures, with 54% of the subjects developing a posttraumatic osteoarthritis at 8-years follow-up. Besides proprioceptive deficit and injury-dependent cartilage and meniscus damage, other reasons for increased rates of osteoarthritis and delayed bone healing may be related to inadequate fracture reduction and fixation [25, 26]. In this context, various strategies have been proposed to achieve absolute stability and interfragmentary compression of Hoffa fractures, with screw fixation representing the most common internal fixation method [2, 6, 22, 42]. However, the current literature lacks reliable evidence regarding proper fixation of comminuted lateral Hoffa fractures, which are occasionally observed in clinical practice [38].

In Hoffa fractures with large posterior condylar fragments (Letenneur type I), recent biomechanical studies have shown that additional plate fixation counteracts vertical shear forces across the fracture site and improves fracture retention [27, 33]. Pires et al. [27] have demonstrated in a synthetic bone model that combined fixation of large Letenneur type I fractures with PA orientated 3.5 mm screws and a 3.5 mm posterior plate resisted higher failure loads (1938 N) than isolated anteroposterior screw fixation (501 N). In their study, a lateral plate-augmented screw fixation exhibited remarkably lower failure loads of 1342 N compared to a posterior plate. However, in these Letenneur type I fractures, the stability of the additional lateral plate fixation might be improved when locking screws are used. In 2017, Sun et al. [33] have shown in a synthetic bone model that the combination of a 6.5 mm PA screw with lateral locking plate fixation provided nearly comparable biomechanical stability to posterior plate augmentation with a failure load of 1710 N. However, the aforementioned studies only considered large Letenneur type I fractures [27, 33] and did not investigate fixation techniques for smaller Letenneur type II Hoffa fractures with their frequently observed intercalated fragments of the comminution zone in the weight-bearing area of the lateral femoral condyle [38].

In the present study, additional lateral locking and posterior plate fixation provided equivalent biomechanical stability for fixation of the Letenneur type II Hoffa fragment with comparable cycles to failure and failure loads. However, the failure loads were lower compared to the aforementioned studies, which might be due to the smaller size of the Hoffa fragment. Letenneur type IIb fractures provide less bone stock to anchor the locking screws of the lateral plate and less contact between the posterior femoral condyle and the posterior plate for a proper buttress effect as compared to fractures with a larger condylar fragment. If these lateral-sided coronal plane fractures are not treated with an appropriate fixation strategy that neutralizes the vertical shear forces, the osteosynthesis is likely to fail, as has been demonstrated biomechanically [33, 41]. In contrast to the Hoffa fragment, the stability of the intercalary fragment could only be improved with a lateral locking plate fixation, resulting in significantly higher cycles to failure and failure loads. Thus, lateral locking plate or double plate fixation might be advantageous in cases of severe comminution.

The results of the present study are clinically relevant considering that the knee has to bear up to twice the body weight during normal gait [31, 37]. When the clinical failure criterion of 2 mm axial displacement of the articular surface was reached, a corresponding failure load of over 1000 N was observed for the plate-augmented treatment of the Hoffa and intercalary fragments. In this context, plate augmentation of direct posteroanterior screw fixation and addressing each fragment separately with the biomechanically appropriate plating technique appears to be essential if early functional rehabilitation is aimed to optimize knee function after surgical treatment. Furthermore, the size of the Hoffa fragments and the presence of a central comminution zone have recently been considered as the key determinant for selection of the surgical approach [19, 20, 23]. Due to the underlying anatomy, not only the visualization of the articular surface but also the trajectories for screw fixation and the placement of additional plates are limited approach-specifically [23, 25, 26]. Several studies recommended to treat Hoffa fracture via a lateral parapatellar arthrotomy [6, 18, 39, 40]. However, although knee extension might facilitate the reduction due to ligamentotaxis of the anterior cruciate ligament, lateral collateral ligament and gastrocnemius muscle, this approach requires a hyperflexed knee position for an optimal visualization of the articular surface for anatomic fracture reduction [19, 20]. In contrast, a posterolateral approach facilitates fracture reduction in an extended knee position and provides the option of extending the approach via a lateral femoral epicondyle osteotomy to improve exposure of intercalary fragments [23]. Additionally, this approach provides the necessary trajectories for crossed PA screw fixation and placement of posterior and/or lateral plates [23, 25–27, 33]. Therefore, regardless of the size of the Hoffa fragments, the posterolateral approaches might be ideal for treatment of lateral Hoffa fractures, as fracture exposure and osteosynthesis can be fracture-specifically adjusted and even escalated to double plate fixation in presence of extensive comminution [23]. Individualized surgical treatment strategies based on a proper knowledge of fragment size- and fracture morphology-dependent stability as well as surgical approach-specific screw trajectories and plate configurations might improve functional outcomes and reduce treatment failures.

The present study has several limitations. First, cadaveric knee specimens of advanced age (73.7 ± 10.9 years) were used, which might have not necessarily reflected the bone quality of patients suffering from Hoffa fractures [36]. Nonetheless, the distal femora were assigned to the groups on the basis of BMD to ensure comparability between the different test conditions [9]. Nevertheless, the interindividual differences in BMD might have influenced the screw anchoring and thus led to subtle measurement inaccuracies [1]. Second, the used fracture model only represents a simplification of the partly inhomogeneous fracture patterns of comminuted Letenneur type II lateral Hoffa fractures. Nevertheless, it is characterized by a high reproducibility, which enables reliable conclusions regarding the stability of the additional plate constructs and thus enhances the development of a surgical strategy based on the fracture morphology. Third, Letennuer type II fractures often involve the popliteus tendon and anterior cruciate ligament attachment sites, which may have an impact on fracture stability. However, the fracture model did not take into account the dynamic influence of those ligamentous structures. Last, the biomechanical performance of the different constructs was assessed in 30° knee flexion only, although the posterior femoral condyles are exposed to higher forces in deeper knee flexion [31].

## Conclusion

Plate-augmented posteroanterior screw fixation of comminuted Letenneur type IIb Hoffa fractures provided greater biomechanical stability than isolated posteroanterior screw fixation. While additional lateral or double plate fixation improves the stability of both the intercalary and Hoffa fragment, posterior plating stabilized only the Hoffa fragment.

## Data Availability

No datasets were generated or analysed during the current study.
